# Viewing distance matter to perceived intensity of facial expressions

**DOI:** 10.3389/fpsyg.2015.00944

**Published:** 2015-07-02

**Authors:** Andreas Gerhardsson, Lennart Högman, Håkan Fischer

**Affiliations:** ^1^Stress Research Institute, Stockholm University, StockholmSweden; ^2^Department of Psychology, Stockholm University, StockholmSweden

**Keywords:** perceptual constancy, facial expression, perceived intensity, psychophysical measure, face perception

## Abstract

In our daily perception of facial expressions, we depend on an ability to generalize across the varied distances at which they may appear. This is important to how we interpret the quality and the intensity of the expression. Previous research has not investigated whether this so called perceptual constancy also applies to the experienced intensity of facial expressions. Using a psychophysical measure (Borg CR100 scale) the present study aimed to further investigate perceptual constancy of happy and angry facial expressions at varied sizes, which is a proxy for varying viewing distances. Seventy-one (42 females) participants rated the intensity and valence of facial expressions varying in distance and intensity. The results demonstrated that the perceived intensity (PI) of the emotional facial expression was dependent on the distance of the face and the person perceiving it. An interaction effect was noted, indicating that close-up faces are perceived as more intense than faces at a distance and that this effect is stronger the more intense the facial expression truly is. The present study raises considerations regarding constancy of the PI of happy and angry facial expressions at varied distances.

## Introduction

Emotional facial expressions are a vital part of the human non-verbal communicative system. It helps motivate actions and guide behavior. We are frequently confronted with facial expressions and many aspects of this type of socioemotional communication have been well documented in previous research, such as the ability to discriminate and categorize facial expressions (Etcoff and Magee, 1992; Young et al., 1997; Fugate, 2013), the cultural universality or diversity of facial expressions (Ekman et al., 1987; Jack et al., 2012) and how facial expressions evoke emotions in the perceiver (Wild et al., 2001). However, research into whether capability to interpret socioemotional information is dependent on the ability to recognize emotional facial expressions, regardless of whether seen from an angle (Matsumoto and Hwang, 2011; Skowronski et al., 2014) or from a distance (Du and Martinez, 2011; Guo, 2013), is surprisingly scarce.

When perceiving a familiar object the characteristics of that object are recognized irrespective of the situation in which it is perceived (Kulikowski and Walsh, 1998). For example, when a known object is presented from a rare angle or at a distance we usually perceive it as the same object even though the retinal image differs from our general representation of that object. The ability to estimate, without effort, the true size of objects irrespective of their retinal size is called size constancy (Kulikowski and Walsh, 1998; Wagner, 2012). Furthermore, the size of an object is an important monocular cue to its distance, especially when other distance cues are lacking (Haber and Levin, 2001), and in that size and distance are in this respect two sides of the same coin (Noble et al., 2006). For example, if a familiar object appears smaller than normal a spatial interpretation of the object occurs and it is perceived as being further away (Gogel and Da Silva, 1987). The ability to correctly estimate the distance of a face based on its size has been found at a single neuron level in macaque monkeys (Rolls and Baylis, 1986), and in humans as young as 5 months old (Yonas et al., 1982). Hence, if an object of familiar size is perceived as smaller than normal it will appear as being further away, and an object of familiar size that is perceived as larger than normal will appear closer.

Whilst the theory of perceptual constancy has previously been applied to various areas, it has not, knowingly, been applied to the study of intensity of emotional facial expressions. In that sense, an emotional facial expression, being a highly familiar stimulus, would be perceived the same invariant of its distance from the viewer. However, unlike many other objects previously studied, emotional facial expression serves a functional purpose, in that it communicates the need to act (Davidson, 1992; Shariff and Tracy, 2011). Such affective signals will in turn have an impact on the behavior of the person perceiving it, (Seidel et al., 2010) and in turn, alter their perception of distance between themselves and the facial expression (Cole et al., 2013). Moreover, how we perceive a facial expression has been found to be directly affected by its emotional content (Phelps et al., 2006). Given that emotional facial expressions serve a motivational purpose a higher level of perceived intensity (PI) could influence an otherwise expected constancy.

The ability to detect and discriminate between facial expressions is well investigated (Ekman et al., 1987; Young et al., 1997; Fugate, 2013). However, in real life, while facial expressions constantly are perceived at different distances, research has so far mostly focused on high intensity expressions presented in a constant, full size view. Thus, the perception of facial expressions from varied distances is under-investigated. Hager and Ekman (1979) employed a discrimination task with observers placed 30, 35, 40, and 45 meters from the facial expression. They discovered that, although accuracy declined, information about the emotion expressed was identifiable at a distance of up to 45 m. Accurate categorization of facial expression has been found to be dependent on the type of expression being displayed (Smith and Schyns, 2009; Du and Martinez, 2011, 2013; Guo, 2013). Du and Martinez (2013) demonstrated differences in the ability to correctly recognize emotional facial expressions both in response time and due to image resolution. Of the basic emotions, happiness and surprise were the only expressions that were recognized in the lowest resolution condition. This is explainable given the more easily recognizable cues of these emotions, such as an open mouth and widened eyes. Similar results were noted in a study peeling of higher spatial frequencies as a way of manipulating the perception of increased distance (Smith and Schyns, 2009). Simulating a viewing distance that ranged from 3.3 to 105.6 m Smith and Schyns found variations in detection sensitivity for different facial expressions and the diagnostic information available via spatial frequency. Although type of expression matters for categorization accuracy the overall effect of size does not seem to have an effect on discriminative ability, that is when the same expression of the same intensity is presented in different sizes (Lee et al., 2006; Guo, 2013). Taken together previous research using size or image resolution as distance manipulations of emotional facial expressions have all focused on categorization of expressions. To our knowledge, no previous research has investigated the PI of emotional facial expressions when presented in different sizes. Unlike previous research this study’s focus lies within the range of close sociable distance (Hall, 1969) (65–208 cm) where nuances of socioemotional communication can be expected to be easily observable. The close distance is considered to be moderately uncomfortable for the observer (Hayduk, 1983).

Perceptual constancy is a well established concept that has been observed in research studying categorization of discrete emotional facial expression (Hager and Ekman, 1979; Lee et al., 2006; Smith and Schyns, 2009; Du and Martinez, 2011; Matsumoto and Hwang, 2011; Guo, 2013; Skowronski et al., 2014). The main aim of the present study was to investigate whether the concept of perceptual constancy also applies to the perception of intensity in the emotional facial expression. That is, how the PI of the emotion corresponds with the manipulated physical intensity when the apparent distance of the face changes.

Facial size was manipulated to imitate viewing distance. Intensity of emotional facial expression was manipulated in order to distinguish a potential effect of emotion expression intensity from a general effect of picture size. PI was measured using the Borg CR100 scale (Borg and Borg, 2001) and as a control measure for the intensity manipulations of the angry and happy faces Perceived Valence (PV) was obtained using a visual analog scale.

## Materials and Methods

### Participants

A total of 71 subjects (42 females) with a median age of 24 (*range 19–50*) years [male 24 (*range 21–43*) years, female 24 (*range 19–50*) years] volunteered to participate and received points as part of a final course grade requirement. Before the experiment all participants were informed according to the code of ethics and signed a written informed consent prior to the study. The participants were not made aware of the true purpose of the study but were informed about a website where briefing would occur later. All reported normal or corrected to normal visual acuity. Participants were recruited via posters at the Department of Psychology, Stockholm University.

### Materials

#### Control Measures

Since some studies have found that mood can have an influence on emotion perception (Niedenthal et al., 2000; Schmid and Schmid Mast, 2010), it was deemed necessary to also measure participants’ current mood state. Hence all participants completed a Swedish translation of The Positive and Negative Affect Scales (PANAS; Watson et al., 1988). The PANAS scale is well-validated, reliable measures of two important affective states (positive and negative). In order to measure, and control for, participants’ general ability to recognize emotions subjects were also asked to completed a Swedish version of the twenty-item Toronto Alexithymia Scale (TAS-20; Simonsson-Sarnecki et al., 2000).

#### Facial Expressions

The face expression stimuli were derived from the Averaged Karolinska Directed Emotional Faces (AKDEF: Lundqvist and Litton, 1998). The AKDEF is a 8-bit grayscale set of pictures of six different emotional expressions (happy, angry, afraid, disgusted, sad, and surprised). The neutral, happy, and angry faces photographed from a straight angle were used in the present study. Each facial expression is depicted by a female and a male face averaged from 35 subjects each and photographed from a distance of approximately 3 meters (more details are available on www.facialstimuli.com). Each expression was warped to five different intensities using Norrkross MorphX software, an open-source program that uses morphing algorithms to blend two pictures (Wennerberg, 2011).

**FIGURE 1:**
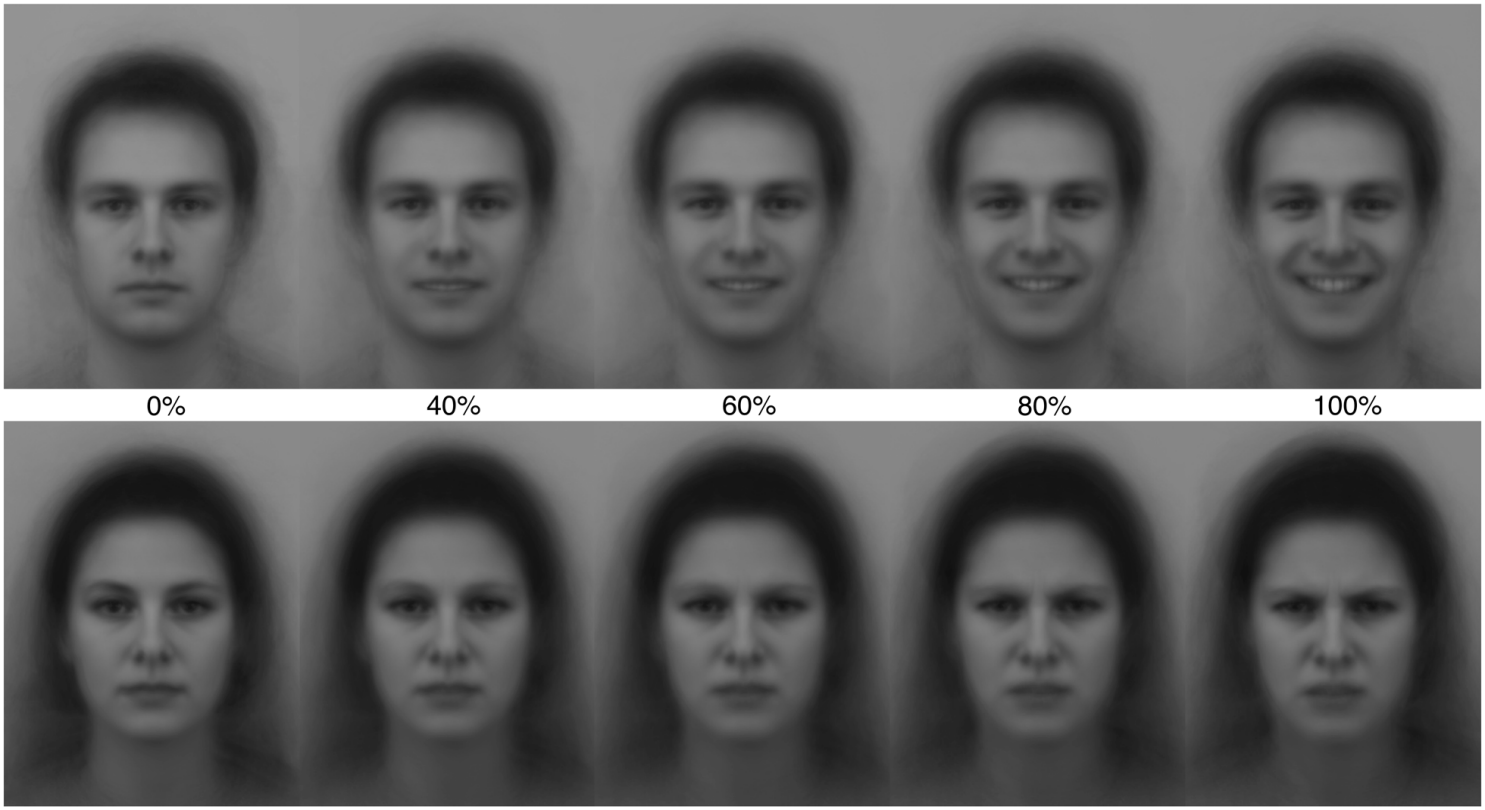
**Examples of the morphed happy male face stimuli and angry female face stimuli ranging from neutral to happy or angry.** Percent figures corresponding to amount of emotional face in blend where 0% is a fully neutral face and 100% is a fully angry or happy face.

Through blending a picture of a neutral face with a picture of an emotional face (happy or angry) six levels of intensity with intermediate morphing steps of 20 were created. The pictures were piloted beforehand. In the pilot the stimuli were shown on a projector in a lecture hall and 133 subjects finished the rating task using the same face stimuli and rating scale as in the present study. As the results of the pilot demonstrated minimal difference of the intensity ratings between 20 and 40%, the 20% level were excluded generating a set of five intensity levels exemplified in **Figure 1**

Each picture stimulus was presented in five different sizes with the original AKDEF size of 19.8 × 26.9 cm used as the largest and corresponding to an almost natural face size of 16 cm (vertical visual angle 13.9°). The scaling was conducted using a factor of 0.75 resulting in the smallest picture having a face length of about 5 cm (vertical visual angle 4.3°). Assuming that size mimics distance, the range between the largest and the smallest face would in terms of distance to the stimuli correspond to approximately 65, 87, 117, 156, and 208 cm from the observer. Distance will be used as a term henceforth. The experiment was built using PsychoPy, an open source, free to use software (Peirce, 2007). The gray background was made to resemble the background of the picture and a raised cosine mask was applied to blur the edges of the picture. All participants used a 24-inch Benq GL2450 s3 monitor, except 4 who, due to technical issues performed the task on a 23-inch Dell 2313H monitor, both LED with a 1920 × 1080 pixel dimension. Screen luminance measured during procedure 60 cm from the screen ranged – depending on face stimuli size – between 34.1 (large face) and 43.7 cd/m^2^ (fixation-cross).

#### Psychophysical Outcome Measures

The PI ratings were obtained using the Borg CR100 scale (Borg and Borg, 2001). The CR100 combines ratio scaling with verbally anchored category levels. It improves the inter-subjectivity of the measure as compared to free magnitude estimation. The CR100 ranges between a value of 0 labeled “Nothing at all” and “Absolute maximum” at above 110. In the present study the scale was computerized and to make it software compatible it was presented horizontally with the low end to the left and high end to the right.

Valence was rated on a horizontal visual analog scale anchored at left (Negative) and right (Positive). The rating output was a continuous value between 0 and 1, the higher the more positive.

### Procedure

Experiments were performed in a normally lit lab (~250 lux) at the Department of Psychology during a period of 3 weeks in the beginning of 2014. A total of five experiment sites were set up with padded screens separating the sites enabling up to five participants simultaneously performing the task. Before each experimental round, participants were briefly introduced to the task at hand. Participants were then told to sit comfortable in front of the monitor and remain at a set position measured 60 cm from the forehead. Chin rests were deemed too uncomfortable for the trial length (~25 min).

**FIGURE 2:**
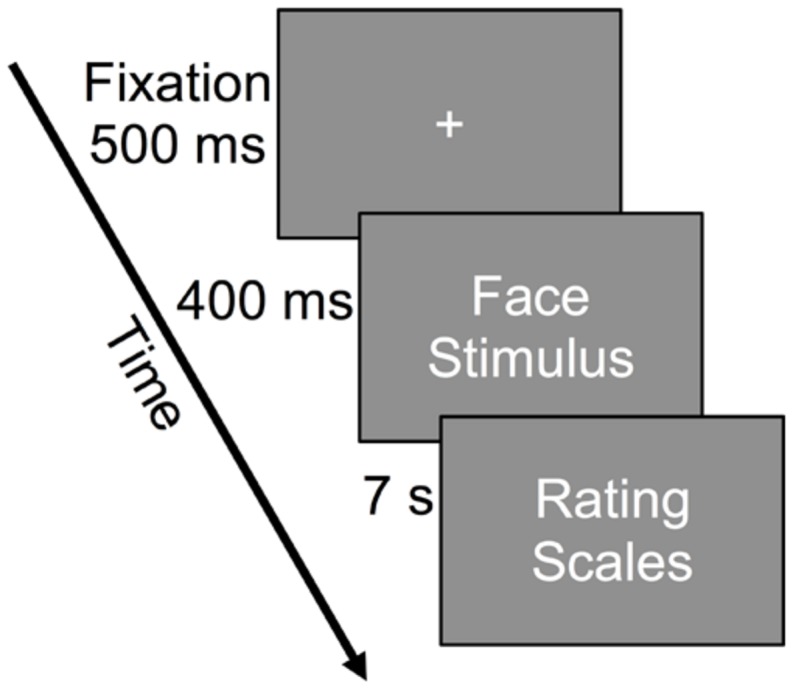
**Experimental routine example.** The face stimuli (varying in gender, expression, intensity, size, and position) were randomly presented.

Participants began by completing the PANAS and TAS-20. After that the valence scale and the Borg CR100 intensity scale were presented with written instructions and participants were able practice and to familiarize themselves with the two scales in their own pace. During this period they were allowed to ask questions. After the familiarization period, a practice trial similar to the experimental trial of six stimuli was assessed. The size manipulation was not used in the practice trial. Instructions were presented on the monitor before the practice trial and the following experimental session asking participants to be spontaneous in their judgments. The experimental sessions consisted of 90 stimuli randomly varying in facial-gender, facial expression and intensity of expression. Each stimulus was presented twice with a minor change in lateral position of a 1° visual angle (left or right) from the center. Although there has been evidence of a lateralization effect (Jansari et al., 2000), no effect of positioning was expected due to the minor degree of this positioning. Each routine consisted of the following (See also **Figure 2**): First a fixation-cross appeared at the center for 500 ms, after that a face stimulus appeared for 400 ms, enough time not to affect detection ability as opposed to no time limit (Calvo and Lundqvist, 2008). In the final judgement stage, the valence scale was displayed at the top of the screen and the horizontal Borg CR100 scale at the bottom of the screen. A thin white line separated these scales and the response time was limited to 7 s to enforce spontaneous judgment. Once this sequence was completed, the processes began again and looped for all 180 stimuli.

### Data Analysis

All statistical analyses were performed using SPSS software, version 22.0. The study was not originally designed for use of covariates and due to adverse effect of power these measures are reported separately. To balance out the analysis between happy and angry faces, neutral faces (Manipulated Intensity 0%) were not used in the analysis.

## Results

As expected lateral positioning of the stimuli (left or right) had no effect on the rating (*p* > 0.10). The PANAS positive and negative subscales were made into a single ratio variable and a Pearson product-moment correlation with the valence scores of angry (*r* = 0.09, *p* = 0.46) and happy faces (*r* = 0.07, *p = 0.55*) did not yield any significant results. For the TAS-20 a Pearson product-moment correlation with valence means of angry (*r* = 0.11, *p* = 0.36) or happy faces (*r* = 0.05, *p* = 0.69) respectively, showed no significant results.

Two sets of 4 × 5 × 2 × 2 repeated measures analyses of variances were conducted, with Manipulated Intensity (four levels: 40, 60, 80, and 100%), Distance [five levels (cm): 65, 87, 117, 156, and 208], Face–Gender (two levels: male and female), and Expression (two levels: angry and happy) as within subject variables where Face–Gender and Expression mainly functioned as control variables. Analyses were performed separately on the dependent variables PV and PI. PV was expected to be tightly associated with PI as only two expressions were used, for example higher Manipulated Intensity was expected to yield a higher PI and a PV score closer to the happy or angry anchor, respectively. Therefore PV was mainly treated as a control measure of the stimuli. Greenhouse–Geisser corrections to the degrees of freedom were applied where sphericity assumption was violated. Alpha level was set on 0.05 for all statistical analyses.

**FIGURE 3:**
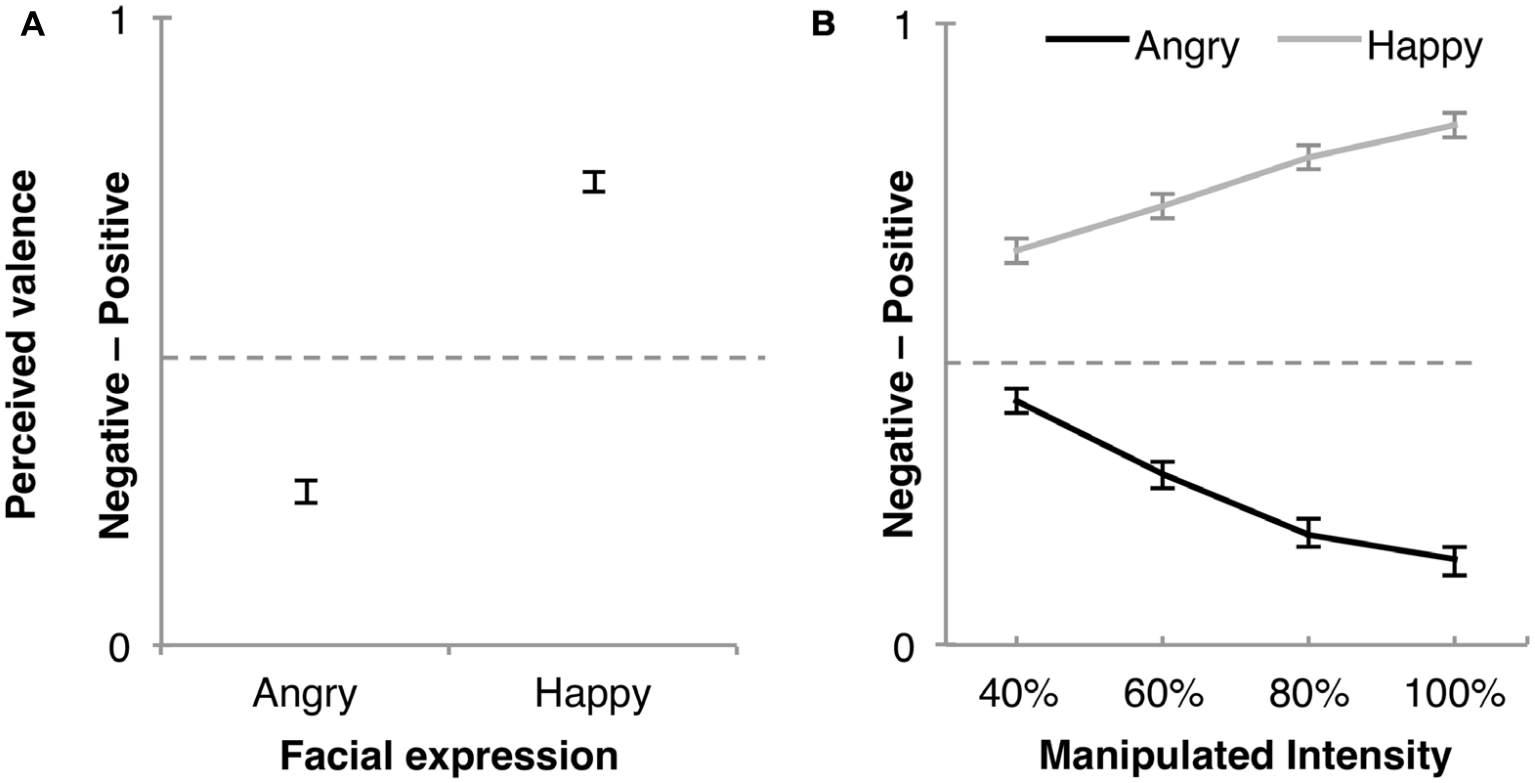
**Middle dotted line represent neutral (0.5). (A)** Angry and Happy expression showing a difference in valence ratings and (B) the Manipulated Intensity × Expression effect indicate that the higher intensity the further away from neutral for Happy and Angry, respectively. Error bars represent ±95% confidence interval.

In a first step PV was used as dependent variable. There was a main effect of Expression *F*(1,70) = 1157.01, *p* < 0.0001, $\eta_{p}^{2}$ = 0.94, and an Expression × Manipulated Intensity interaction *F*(1.51,105.72) = 342.77, *p* < 0.0001, $\eta_{p}^{2}$ = 0.83, indicating that Happy was perceived positive, Angry was perceived negative, both showing a greater effect with increasing Manipulated Intensity (**Figure 3**). A main effect was also found for Gender *F*(1,70) = 33.47, *p* < 0.0001, $\eta_{p}^{2}$ = 0.32 showing that female faces were in general more positively rated than male faces. Additionally, there was a significant Distance × Expression interaction *F*(3.33,232.87) = 5.68, *p* = 0.0006, $\eta_{p}^{2}$ = 0.08, and a Distance × Intensity × Expression interaction *F*(6.79,475.61) = 2.16, *p* = 0.039, $\eta_{p}^{2}$ = 0.03 both mainly driven by the Expression effect.

**FIGURE 4:**
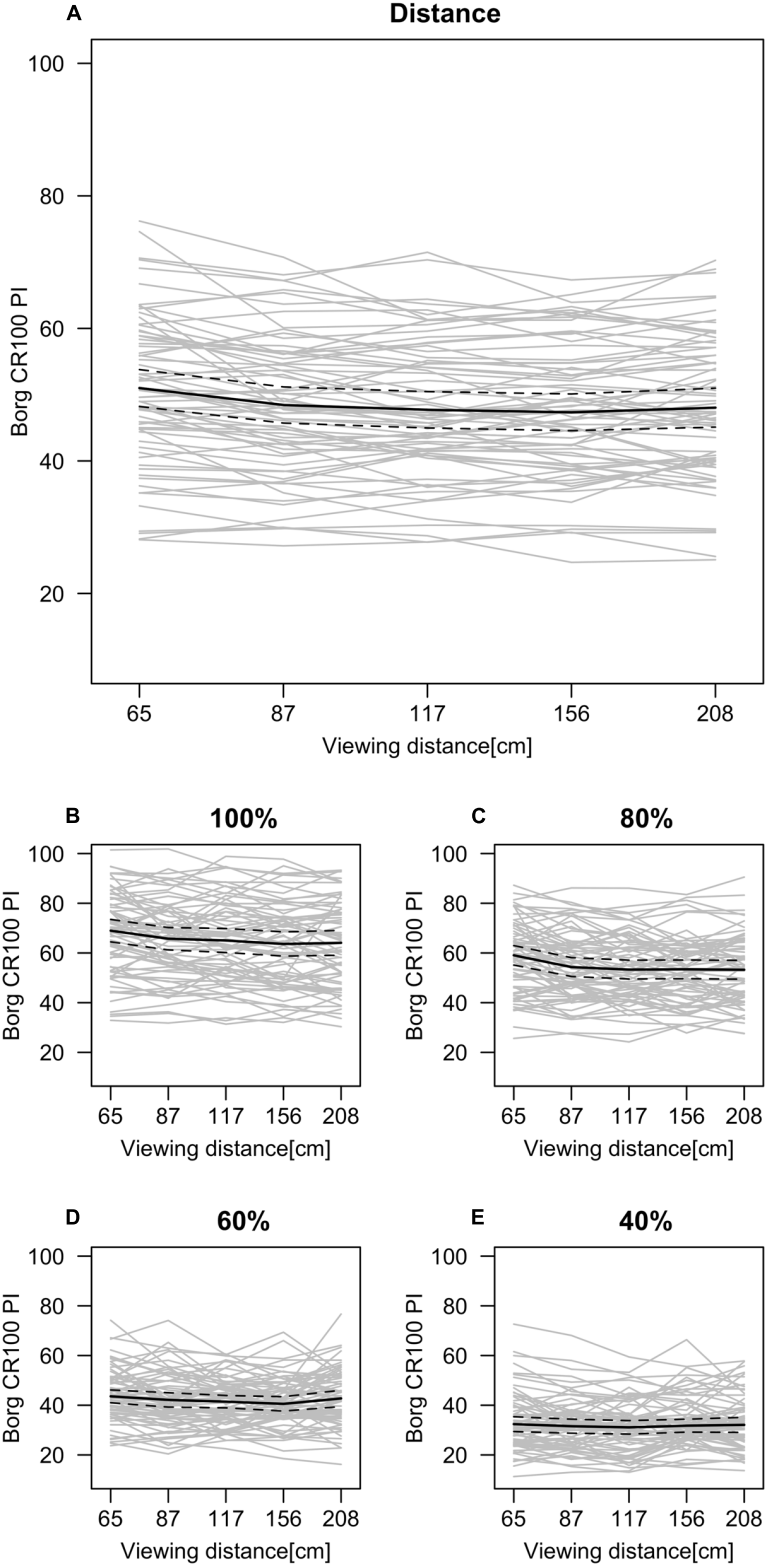
**Marginal mean estimate (black line) of PI on effects of Distance (A) and Distance×Manipulated Intensity interaction on separate plots (B–E) indicating higher intensities to have a more positive linear trend than lower intensities.** Gray lines represent each subject’s mean. Dotted lines represent ±95% confidence interval.

In the second analysis PI was used as dependent variable. First of all, a significant main effect of Manipulated Intensity was evident *F*(1.25,57.59) = 151.87, *p* < 0.0001, $\eta_{p}^{2}$ = 0.77, with a significant linear contrast *F*(1,46) = 171.34, *p* < 0.0001, $\eta_{p}^{2}$ = 0.79, and cubic contrast *F*(1,46) = 9.10, *p* = 0.004, $\eta_{p}^{2}$ = 0.17. Distance had a significant main effect on the PI *F*(1.95,89.82) = 10.30, *p* < 0.0001, $\eta_{p}^{2}$ = 0 0.18, with a signifi-cant linear contrast *F*(1, 46) = 9.08, p < 0.004, $\eta_{p}^{2}$ = 0.17, and a significant quadratic contrast *F*(1,46) = 28.71, *p* < 0.0001, $\eta_{p}^{2}$ = 0.38 (**Figure 4A**). Furthermore, there was a significant Distance × Manipulated Intensity interaction *F*(7.89,362.77) = 2.70, *p* = 0.007, $\eta_{p}^{2}$ = 0.06. A significant linear contrast further qualified the interaction *F*(1,46) = 18.29, *p* < 0.0001, $\eta_{p}^{2}$ = 0.29 (**Figure 4B-E**). No significant main effects were found for face gender *F*(1,46) = 0.04, *p* = 0.85, or face expression *F*(1,46) = 0.34, *p* = 0.57.

## Discussion

Using a psychophysical approach, the purpose of the present study was to manipulate an apparent distance of the faces to investigate the concept of perceptual constancy in relation to the perception of emotional facial expression intensity. The results of the present study indicate that (1) the distance of the face affects the PI of the facial expression and that (2) this effect is stronger for higher intensity manipulations.

Past research has found that discrimination and categorization of different emotional facial expressions are not fully constant when perceived from a distance (Hager and Ekman, 1979; Lee et al., 2006; Smith and Schyns, 2009; Du and Martinez, 2011; Matsumoto and Hwang, 2011; Guo, 2013; Skowronski et al., 2014). Deviations from constancy have also been observed when additional manipulation has been made to the stimuli, for example when presentation duration is short or when resolution is low (Du and Martinez, 2013; Skowronski et al., 2014). The results of the present study complements previous research by showing that the PI of an emotional facial expression is not fully constant to the manipulated physical intensity of the face, but rather is dependent on the perceived distance of the face. The faces appearing closer were perceived more intense than the more distal faces but previous research investigating categorization accuracy found distance (or size) to be invariant (Lee et al., 2006; Guo, 2013). Perception, however, involves more than just detection and categorization. The findings in the present study would indicate that emotional content of a face can alter how it is perceived (Phelps et al., 2006) and thus, is not constant. According to the perceptual constancy phenomenon, distance or size should not affect the perception of a familiar object. However, as facial expressions are communicative displays of socioemotional information, the closeness of the face at a distance of 65 cm appears to have affected the PI of the emotion. The conception that emotion influences perception was further supported by the interaction of distance and manipulated intensity. If the effects were to depend on the increased vividness and detail of the face, the increase in PI would be evident at all manipulated intensity levels (see **Figures 4B–E**). While there was no significant effect of distance on a face where the emotional intensity was less intense (40 and 60%), the PI was more affected by the distance of the face when the expression intensity increased (80 and 100%).

The goal in this study was to keep the face within a communicative sociable distance, each level decreases with a factor of 0.75 with the furthers face appearing at a distance of 2.08 m, compared to other studies using a factor of 0.25 with the distal face at 5 m (Guo, 2013) and a factor of 0.5, distal face at 105.6 m (Smith and Schyns, 2009). The estimated marginal means of distance all corresponded approximately to verbal anchor “Strong” and the small effect could be explained by the small difference between the distance measures. As an alternative to increasing distance future research should try a different warping manipulation of the faces, as perception of a three-dimensional object being further away changes in other ways than the just a general increase in retinal size (see Bryan et al., 2012). This would further emphasize the effect of a close-up face and possibly strengthening the effect.

Given that emotional expression triggers action, a face perceived as being closer might promote a greater urge to react than a face seen from a greater distance. It has been well established that threatening stimuli is preferably kept at a distance from the viewer, than non-threatening stimuli (Marsh et al., 2005; Seidel et al., 2010; Stins et al., 2011). However, different expressions can be categorized as serving a distal or close-up purpose (Smith and Schyns, 2009). Smith and Schyns found happiness to be associated with distal communication, whereas anger was associated with more close-up communication. In the present study the happy and angry faces were perceived as positive and negative, respectively, a perception that became heightened in correlation with increased emotional intensity. However, the small stimulus set makes any conclusion about the effect of any specific emotion on PI speculative and the same applies to facial gender. The stimuli set needed to be kept small in order to run the experiment within a reasonable time frame, thus avoiding the effects of fatigue. Using non-averaged faces or adding emotional expressions would have prolonged the experiment time considerably.

The present study attempted a more ecologically valid understanding of face perception, by varying intensity and size of the emotional expressions in order to better resemble real life conditions. However, the stimuli of averaged faces used (AKDEF: Lundqvist and Litton, 1998) were gray-scale, static, and composite of 35 different faces, so they can not be said to be as sharp as a single photo. The morphing attenuates high spatial frequencies, which could have had an effect on distance as well as size perception (Smith and Schyns, 2009). Nonetheless, as they are averaged, the focus can be kept on the emotion and not on other salient facial characteristics.

Intensity ratings were made on a Borg CR 100 scale (Borg and Borg, 2001), which for technical reasons was tilted and presented horizontally. Using this psychophysical scale has, to the best of our knowledge, not been done before. As opposed to free magnitude estimation it improves the inter-subjectivity. Being verbally anchored it also gives a meaningful measure of effect size that can be used compare between studies.

This study was not designed to examine gender differences in the observers and was therefore underpowered in terms of number of included subjects to study this important factor. However, given that facial gender is an important factor that affects perception of faces (e.g., Fischer et al., 2004), future studies with higher power should be implemented to investigate how gender of both sender and observer influence the experience of intensity of emotional faces that vary in size.

Knowing more about how we perceive facial expressions is important not just because a human face is one of our first visual experiences in life, but also because throughout our lifetime we are continually exposed to faces signaling emotional information. In an increasingly complex social environment, it is important to study perceptual abilities beyond identification and categorization. Understanding how the face is perceived and how it changes depending on the distance is useful, for example in all situations where facial expressions are used to induce an emotion or send a message (e.g., in marketing, film, and video games). As distance matters to how the facial expression is perceived a combination of intensity and size adjustment could increase the effect for the observer. To further investigate how constancy interplays with PI of emotional expressions, other manipulations associated with perceptual constancy should be employed. For example, how we perceive an angry face in various light conditions or from different angles and how these factors interact. Or how we perceive full-body, emotional expressions under various conditions such as distance, angle or light. The impact of individual differences related to personality and social anxiety could also be investigated using a psychophysical approach.

## Conclusion

The present study demonstrates that the PI of emotional facial expression does not entirely follow the concept of perceptual constancy. Instead, the PI of the emotional facial expression appears to be dependent on the perceived distance of the face and the intensity of the expression. Furthermore, the results illuminate a need for greater diversity in emotion perception research, in order to be able to reach an understanding beyond categorization ability.

## Conflict of Interest Statement

The authors declare that the research was conducted in the absence of any commercial or financial relationships that could be construed as a potential conflict of interest.
